# Prevalence of thyroid nodule and relationship with physiological and psychosocial factors among adults in Zhejiang Province, China: a baseline survey of a cohort study

**DOI:** 10.1186/s12889-024-19375-z

**Published:** 2024-07-11

**Authors:** Xueqing Li, Zhijian Chen, Lizhi Wu, Pengchen Tu, Zhe Mo, Mingluan Xing

**Affiliations:** Department of Environmental Health, Zhejiang Provincial Center for Disease Control and Prevention, 3399 Binsheng Road, Hangzhou, 310051 China

**Keywords:** Thyroid nodule, Prevalence, Ultrasonography, Physiological factor, Psychosocial factors, Zhejiang Province

## Abstract

**Background:**

Thyroid nodules have attracted much attention due to their high incidence and potential for malignant transformation. Compared with the clinical assessment and diagnosis of thyroid nodules, there are relatively few studies on the epidemiological risk factors for thyroid nodules. The aim of this study was to investigate the prevalence of thyroid nodule among adults in Zhejiang province and to explore their relationship with physiological and psychosocial factors.

**Methods:**

The data used in this study were obtained from the baseline survey of the Zhejiang Provincial Cohort Study on Environment and Health. From June 2022 to December 2023, a total of 21,712 participants from five representative cities in Zhejiang were recruited for the baseline survey. Based on the inclusion and exclusion criteria, 15,595 adults were included in the analysis. The data were collected via self-report questionnaires and physical examinations. Multivariate logistic regression analysis was subsequently performed.

**Results:**

The detection rate of thyroid nodules was 50.98% among adults in Zhejiang province. Age, gender, education level, BMI, tea and alcohol consumption all had a statistically significant association with thyroid nodules (*p* < 0.05). After adjusting for sociodemographic factors, results of logistic regression analysis showed that good life satisfaction (OR = 0.854, 95% CI: 0.780–0.934) had a lower risk of thyroid nodules, however, poor life satisfaction (OR = 1.406, 95% CI: 1.014–1.951), social isolation (OR = 1.294, 95% CI: 1.089–1.538) and a family history of thyroid nodules (OR = 1.334, 95% CI: 1.064–1.672) had a greater risk of thyroid nodules.

**Conclusion:**

The detection rate of thyroid nodules in adults of Zhejiang province was an increasing trend compared with that in previous years. In addition to the sensitive thyroid nodule screening technology, influencing factors mentioned in this study might also represent credible candidates for this increase. As variable influence factors, weight management, good interpersonal relationships and life satisfaction should be the focus of health interventions.

**Supplementary Information:**

The online version contains supplementary material available at 10.1186/s12889-024-19375-z.

## Background

The global incidence of thyroid disease is severe and continues to increase. Among thyroid diseases, thyroid nodules (TNs) are the most common, and the prevalence of TNs varies in different countries due to different detection methods (palpation, ultrasound, or autopsy), ranging from 20 to 76% in the general population [1, 2]. The pathological progression of TNs is relatively indolent, but approximately 5–15% of thyroid nodules eventually become malignant. Even so, identification of a nodule generates anxiety for both patients and clinicians regarding the risk for neoplasia. The reported incidence of thyroid cancer varies significantly worldwide, and the highest incidence rates were found in Eastern Asia [3–5]. Between 1998 and 2012, the age-standardized rates (ASRs) of thyroid cancer in both men and women were highest in Korea, far exceeding those in China and Japan [6]. The incidence in Korea has shown decreasing trends since 2013, and improvements in overdiagnosis rates are considered to be the main reason for such a sudden decline [7]. If overdiagnosis was the sole reason for the global increase in the incidence of thyroid cancer, mortality would be expected to decline, as diagnosed patients would receive more appropriate treatment earlier and avoid disease-related death. However, this finding is not consistent with the increasing incidence and mortality of thyroid cancer [4]. Increasing evidence has shown that environmental exposure and modifiable risk factors are also responsible for the increase in incidence [4, 8, 9].

Negative emotions are closely related to the occurrence and development of many diseases, especially endocrine system diseases [10]. TNs represent a pathological change resulting from local abnormal growth of thyroid cells and are a common and frequently occurring disease of the endocrine system. Therefore, from the perspective of psychosomatic medicine, persistent negative emotions are likely related to thyroid nodules [11, 12]. In addition, studies have shown that negative emotions can damage immune function [13], and a disorder of immune function leads to autoantibodies constantly attacking thyroid cells, leading to not only thyroid dysfunction but also abnormal proliferation, division and replication of thyroid cells, the formation of thyroid nodules, and even thyroid cancer [14]. Therefore, recently, a large number of studies have been conducted to explore the pathogenesis and prognosis of thyroid diseases from a psychological perspective [15]. The associations between anxiety, depression, stress, poor sleep quality and thyroid nodules have gradually attracted the attention of scholars worldwide [11, 16]. Compared with those of psychological factors, the associations of age, gender, BMI, smoking status, drinking status, and radiation exposure with the risk of thyroid diseases have been reported, but the effects of drinking status and radiation exposure are still controversial [11, 17]. Moreover, compared with heated discussions and in-depth studies on the evaluation and diagnosis of thyroid nodules [18], epidemiological investigations of the associated risk factors are insufficient. However, the concept of prevention before treatment needs to be emphasized and implemented, whether from the perspective of avoiding physical and mental health distress or reducing social and economic burdens.

Zhejiang is an economically developed province on the east coast of China in which the salt iodization program has been implemented earlier. However, there are few reports on the prevalence of thyroid nodules in the general population in Zhejiang Province and the influence of factors other than iodine. We aimed to identify effective paths and reasonable measures for future health interventions in Zhejiang Province by exploring the associations of thyroid nodule prevalence with sociodemographic factors, social psychological status, exogenous substance exposure and family history, and the association between individual sleep status and thyroid nodule prevalence in the general population.

## Methods

### Participants

**Fig. 1:**
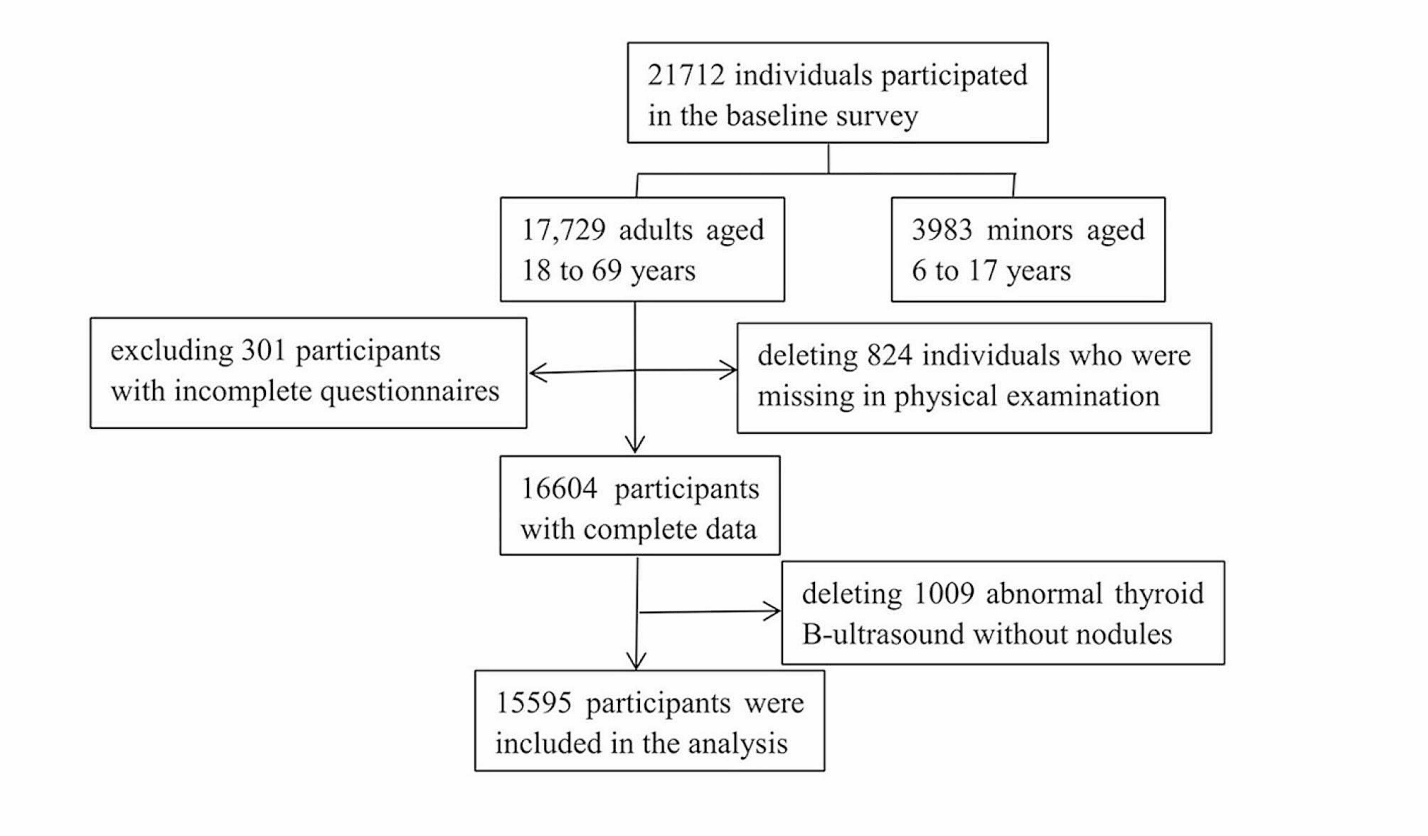
Flowchart of selecting participants in this study

The data used in this study were obtained from the baseline survey of the Zhejiang Provincial Natural Population Cohort Study on Environment and Health. The entire research framework of the study is shown in Fig. 1. Based on the inclusion and exclusion criteria, eligible participants were enrolled from five representative cities in Zhejiang, namely, Huzhou (north), Quzhou (west), Jinhua (central), Taizhou (east), and Lishui (south), between June 2022 and December 2023. The recruitment strategy for the study participants was as follows: (1) 3–5 subdistricts or towns were selected randomly from each city; (2) 5 communities or villages located in the east, west, south, north and middle were selected from each subdistrict or town; and (3) 100–300 individuals from each community or village were randomly selected. The inclusion criteria were as follows: (1) local resident population aged 6–69 years; (2) conscious and able to communicate normally; and (3) signed informed consent. The exclusion criteria were as follows: (1) had cancer; (2) had a mental illness or cognitive impairment; (3) had received any iodine-containing drugs or contrast agents in the previous three months; and (4) refused to cooperate. In this study, only adults were selected. All minors (aged < 18 years, *n* = 4441), adults with incomplete questionnaires or improbable answers (301), those with missing physical examination data (824) and those without thyroid nodules but with abnormal thyroid B-ultrasound results (1009) were excluded. Ultimately, 15,595 adults (age ≥ 18 years) were included in the analysis, and the response rate was 87.96%.

### Data collection

The data were collected by professional technicians who had been well trained using self-report questionnaires and physical examinations. The self-report questionnaires consisted of four sections: demographic characteristics, social psychological status, exogenous substance exposure and family history, and sleep assessment (Table S1 and S2), which were designed by the project team on the basis of a literature review and previous experience and were determined after expert discussion and evaluation.

The demographic characteristics included age, gender, height, weight, education level, smoking status, alcohol and tea consumption status. Height and weight were measured using uniform equipment. Body mass index (BMI) was calculated as weight (kg) divided by height squared (m^2^). BMI was classified into four categories according to WHO-approved guidelines: “underweight”, less than 18.5 kg/m^2^; “normal weight”, between 18.5 and less than 25.0 kg/m^2^; “overweight”, between 25.0 kg/m^2^ and less than 30.0 kg/m^2^; and “obesity”, between 30.0 kg/m^2^ and above.

The social psychological indicators were based on previous reports [19–22] and consisted of the following seven questions: “Are you satisfied with your life [19]? ”, “Have you experienced any of the following severe events during the past two years [20]? ”, “Have you felt depressed in the last month [21]? ”, “Have you felt nervous or anxious in the last month?”, “Do you often feel the lack of company?” [22], “Do you often feel isolated from others?” and “Do you often feel ignored?”. There were three options for the first question: (1) dissatisfied, (2) generally satisfied, and (3) satisfied. The second question consisted of the following 10 items: separated/divorced, unemployed/laid-off/retired, economic bankruptcy of self- or family-owned business, violently attacked/raped, serious internal family conflicts and clashes, severe trauma or traffic accidents, death of spouse, another family member died or became seriously ill, severe natural disasters (e.g., drought, flood, etc.), and loss of income/living in debt. Participants were divided into two categories based on whether they experienced the aforementioned events. For the last five questions, yes or no answers were given.

In terms of exogenous substance exposure and family history, four questions were designed to collect information [23, 24]. (1) Do you take any iodine supplements in addition to food and salt? (2) Have you taken any of the following medications in the last three months? amiodarone, phenytoin sodium, rifampicin, estrogen, androgen, somatotropin, long-acting contraceptives, heparin. (3) Have you received any of the following examinations or treatments for your head and neck in the past three years? Radionuclide Co 60 in the neck, I 131 radiation in the neck, CT examination of the neck, magnetic resonance imaging of the neck. (4) Did your parents or siblings have a diagnostic history of thyroid nodules? Responses to the first three questions were dichotomized as yes or no. In addition to yes or no, the third possible answer to the last question was “unclear”.

The sleep assessment included the Pittsburgh Sleep Scale [16, 25] and three questions related to sleep. The Pittsburgh Sleep Quality Index (PSQI) was used to assess sleep quality and disorders. It is a self-report questionnaire and contains seven component scores: subjective sleep quality, sleep latency, sleep duration, habitual sleep efficiency, sleep disturbance, use of sleeping medication, and daytime dysfunction. The score range of each question is 0–3, and the total score ranges from 0 to 21. The higher the score is, the worse the sleep quality. The cutoff points were as follows: scores greater than or equal to 5 points indicated poor sleep quality, and scores less than 5 points indicated good sleep quality [25]. Three questions are as follows: In your current or previous job, did you often work night shifts? Do you have the habit of taking a nap? Have you ever been diagnosed with obstructive sleep apnea?

In this study, physical examinations, even height and weight, were performed by clinical practitioners. Ultrasound investigations of the thyroid were performed by one registered physician who had a professional certificate for ultrasonography (awarded by the Ministry of Health of China) and one assistant. Ultrasound was performed using MicroMaxx portable color Doppler ultrasound diagnostic instruments (FUJIFILM SonoSite, Inc., Washington, USA) with a transducer probe frequency of 7.5 MHz. The participant was positioned in a supine position and the head tilted back as far as possible to fully expose the thyroid gland. A thyroid nodule was defined as an additional structural focal abnormality found by ultrasound that was radiologically distinct from the surrounding thyroid parenchyma. For data analysis, participants were divided into a thyroid nodule group (TN group) and a no-thyroid nodule group (no-TN group) according to the presence or absence of thyroid nodules.

### Statistical analyses

IBM SPSS version 25.0 (IBM Corporation, New York, NY, USA) was used for data processing and statistical analyses. Data with missing values for any variables (301 participants with incomplete questionnaires and 824 participants lacking physical examination data) were excluded from the analysis. Valid data were described and analyzed by using frequencies, percentages and mean ± SD.

The variables were transformed into classification grades according to the principles described in the methods section, and then the chi-square (χ2) test and binary logistic regression analysis were performed on the classification variables. Potential risk factors (*p* < 0.05) from the chi-square (χ2) test were included in the multivariate stepwise logistic regression analysis (forward LR), and the p values for the entry and removal of variables in the stepwise logistic regression model were 0.05 and 0.1, respectively. The results are expressed as odds ratios (ORs) and 95% confidence intervals (CIs). All tests were two-tailed cutoffs, and significance was set at the 0.05 level.

### Ethics

#### Ethical approval

was obtained from the Ethics Committee of Zhejiang Provincial Center for Disease Control and Prevention (approval no. 202,004,001). Written informed consent was obtained from all participants during the study period. Participants were allowed to withdraw from the study at any time. Abnormal baseline survey and physical examination results were evaluated by clinicians in a timely manner. Potential patients were given feedback and professional advice face-to-face or via telephone.

## Results

### Sociodemographic characteristics of the participants

A total of 21,712 participants were recruited for the baseline survey. In this study, we only focused on the prevalence of thyroid nodules and the influence of physical and mental factors in adults in Zhejiang Province; therefore, 15,595 participants were ultimately included in the data analysis according to the exclusion criteria described in the Methods section. The sociodemographic characteristics of the population are shown in Table 1. The mean age of the participants was 51.67 ± 12.99 years, of which 18–34 years accounted for 14.06%, 35–59 years accounted for 53.79%, and 60–69 years accounted for 32.16%. In terms of gender distribution, the proportion of females was relatively high, accounting for 62.34%. The education level of the respondents was mostly junior high school and below, accounting for 70.89%. According to the BMI, 60.47% of the participants were normal weight, 30.68% were overweight, and less than 10% were underweight or obese. The percentages of people who did not drink tea, did not drink alcohol and did not smoke were 74.79%, 80.35% and 85.32%, respectively. The differences in all the above variables between the two groups of people with and without thyroid nodules according to the chi-square test were significant (*p* < 0.01).

**Table 1:** The sociodemographic characteristics of the participants

Variable	group	total	no-TN group	TN group	*p*
		*n* (%)	*n* (%)	*n* (%)	
Age (y)		51.67 ± 12.99	48.83 ± 13.41	54.40 ± 11.95	**< 0.001**
Age groups	18–34	2192 (14.06%)	1433 (18.75%)	759 (9.55%)	**< 0.001**
	35–59	8388 (53.79%)	4330 (56.65%)	4058 (51.04%)	
	60–70	5015 (32.16%)	1881 (24.61%)	3134 (39.42%)	
Gender	male	5873 (37.66%)	3409 (44.60%)	2464 (30.99%)	**< 0.001**
	female	9722 (62.34%)	4235 (55.40%)	5487 (69.01%)	
Education level	junior high school or below	11,055 (70.89%)	5047 (66.03%)	6008 (75.56%)	**< 0.001**
	high school	2357 (15.11%)	1252 (16.38%)	1105 (13.90%)	
	college or above	2183 (14.00%)	1345 (17.60%)	838 (10.54%)	
BMI	18.5–24.9 kg/m^2^	9430 (60.47%)	4695 (61.42%)	4735 (59.55%)	**< 0.001**
	< 18.5 kg/m^2^	559 (3.58%)	329 (4.30%)	230 (2.89%)	
	25–29.9 kg/m^2^	4784 (30.68%)	2236 (29.25%)	2548 (32.05%)	
	≥ 30.0 kg/m^2^	822 (5.27%)	384 (5.02%)	438 (5.51%)	
Tea	no	11,663 (74.79%)	5509 (72.07%)	6154 (77.40%)	**< 0.001**
	yes	3932 (25.21%)	2135 (27.93%)	1797 (22.60%)	
Alcohol	no	12,531 (80.35%)	5908 (77.29%)	6623 (83.30%)	**< 0.001**
	yes	3064 (19.65%)	1736 (22.71%)	1328 (16.70%)	
Smoking	no	13,305 (85.32%)	6307 (82.51%)	6998 (88.01%)	**< 0.001**
	yes	2290 (14.68%)	1337 (17.49%)	953 (11.99%)	

Note: Data are expressed as the mean ± SD or n (%). Abbreviations: BMI, body mass index; no-TN group, no thyroid nodule group; TN group, thyroid nodule group. Bold fonts indicate statistically significant differences

### The rate of thyroid nodules examined by thyroid B-ultrasound in this study

A total of 15,595 participants were included in the final analysis. By ultrasound, professional doctors found that 7951 individuals had thyroid nodules. The detection rate of thyroid nodules was 50.98% in the study.

### Comparison of the distribution of various factors between the thyroid nodule and no-thyroid nodule groups

The distributions of various factors, such as social psychological status, life exposure, family history and sleep assessment status, between the thyroid nodule and no-thyroid nodule groups are listed in Table 2. The chi-square test was used to compare the factors between the two groups. Regarding social psychology, the differences between the two groups in terms of life satisfaction, experiencing severe negative life events, experiencing depression and being isolated by others were significant (*p* < 0.01). In the case of exogenous substance exposure and family history, the differences between the two groups were significant in terms of history of neck radiological examination or treatment within the past three years and family history of thyroid nodules (*p* < 0.01). There were also significant differences (*p* < 0.01) in sleep indices, napping habits and frequent night shifts between the groups with and without thyroid nodules. However, the differences between the two groups in the analysis of emotional anxiety, lack of peers and neglected social interaction, history of intake of iodine supplements and thyroid damage medication, and history of apnea were not significant.

**Table 2:** Different distributions of various factors between the no-TN and TN groups

Variable	group	no-TN group	TN group	*p*
		*n* (%)	*n* (%)	
Social psychology				
Life satisfaction	general	6278 (82.13%)	6724 (84.57%)	**< 0.001**
	good	66 (0.86%)	95 (1.19%)	
	poor	1300 (17.01%)	1132 (14.24%)	
Experience of severe life events	no	6512 (85.19%)	6655 (83.70%)	**0.010**
	yes	1132 (14.81%)	1296 (16.30%)	
Anxiety or stress	no	6432 (84.14%)	6611 (83.15%)	0.092
	yes	1212 (15.86%)	1340 (16.85%)	
Depression	no	6356 (83.15%)	6515 (81.94%)	**0.047**
	yes	1288 (16.85%)	1436 (18.06%)	
Lack of peers	no	6974 (91.23%)	7224 (90.86%)	0.408
	yes	670 (8.77%)	727 (9.14%)	
Feel ignored	no	7205 (94.26%)	7446 (93.65%)	0.111
	yes	439 (5.74%)	505 (6.35%)	
Feel isolated	no	7397 (96.77%)	7601 (95.60%)	**< 0.001**
	yes	247 (3.23%)	350 (4.40%)	
Life exposure and family history				
Intaking iodine supplements	no	7597 (99.39%)	7910 (99.48%)	0.408
	yes	47 (0.61%)	41 (0.52%)	
Intaking thyroid damage medication	no	7596 (99.37%)	7902 (99.38%)	0.926
	yes	48 (0.63%)	49 (0.62%)	
History of neck radiation	no	7084 (92.67%)	7281 (91.57%)	**0.011**
	yes	560 (7.33%)	670 (8.43%)	
Family history of thyroid nodules	no	6982 (91.34%)	7111 (89.44%)	**< 0.001**
	yes	155 (2.03%)	177 (2.23%)	
	unclear	507 (6.63%)	663 (8.34%)	
Sleep-related assessment				
PSQI	good	5878 (76.90%)	5823 (73.24%)	**< 0.001**
	bad	1766 (23.10%)	2128 (26.76%)	
Nap habit	no	3206 (41.94%)	3276 (41.20%)	**0.002**
	throughout the year	1955 (25.58%)	1896 (23.85%)	
	at certain seasons	2483 (32.48%)	2779 (34.95%)	
Often work night shifts	no	6869 (89.86%)	7280 (91.56%)	**< 0.001**
	yes	775 (10.14%)	671 (8.44%)	
History of apnea	no	7227 (94.54%)	7532 (94.73%)	0.607
	yes	417 (5.46%)	419 (5.27%)	

Note: abbreviations: no-TN group, no thyroid nodule group; TN group, thyroid nodule group; PSQI, Pittsburgh Sleep Quality Index. Bold font indicates statistically significant differences

### Correlations between depressive symptoms and various factors

The relationships between the aforementioned individual factors and thyroid nodules were analyzed through chi-square tests, and statistically significant variables were selected for multifactor logistic regression analysis. The specific results are presented in Table 3. Sociodemographic factors were not considered in Model 1, while they were adjusted in Model 2 based on Model 1. After incorporating the correction factors, several factors, such as history of severe negative life events, neglected social interaction, neck radiation history, poor sleep index, and frequent night shifts, were excluded from the equation by logistic regression analysis. The impact of these factors on thyroid nodules may be strongly influenced by sociodemographic factors. Finally, multivariate logistic regression analysis indicated that age, gender, education level, BMI, tea and alcohol consumption all had a statistically significant association with thyroid nodules (*p* < 0.05). The risk of thyroid nodules was greater in people aged 35–59 years and 60–69 years than in people aged 18–34 years. The ORs were 1.606 (95% CI: 1.431–1.804) and 3.060 (95% CI: 2.692–3.479), respectively. Females had a greater risk of thyroid nodules than males (OR = 1.864, 95% CI: 1.727–2.011). Compared with participants with a junior high school education or below, those with a college education or above had a lower risk (OR = 0.860, 95% CI: 0.757–0.977). Using body mass index (BMI) as an indicator of body weight, compared with normal weight, the ORs of underweight, overweight and obesity were 0.744 (95% CI: 0.621–0.891), 1.164 (95% CI: 1.082–1.252) and 1.278 (95% CI: 1.102–1.483), respectively. Tea (OR = 0.885, 95% CI: 0.818–0.957) and alcohol (OR = 0.842, 95% CI: 0.768–0.923) had a similar risk of thyroid nodules. After adjusting for the above factors, results showed that satisfaction with life was positively correlated with the risk of thyroid nodules. Good life satisfaction (OR = 0.854, 95% CI: 0.780–0.934) and poor life satisfaction (OR = 1.406, 95% CI: 1.014–1.951) have different risk of thyroid nodules from general life satisfaction. In addition, social isolation (OR = 1.294, 95% CI: 1.089–1.538) and a family history of thyroid nodules (OR = 1.334, 95% CI: 1.064–1.672) were also influencing factors of thyroid nodules. Finally, compared with participants didn’t nap, those with a habit of napping only at certain seasons (but not throughout the year) had a higher risk (OR = 1.150, 95% CI: 1.066–1.240).

**Table 3:** Results of multivariate logistic regression analysis for thyroid nodules

Variable	group	Model 1		Model 2	
		OR (95% CI)	*p*	OR (95% CI)	*p*
Age groups	18–34			1.000 (reference)	
	35–59			1.606 (1.431–1.804)	**0.000**
	60–69			3.060 (2.692–3.479)	**0.000**
Gender	male			1.000 (reference)	
	female			1.864 (1.727–2.011)	**0.000**
Education level	junior high school or below			1.000 (reference)	
	high school			1.061 (0.964–1.167)	0.228
	college or above			0.860 (0.757–0.977)	**0.020**
BMI	18.5–24.9 kg/m^2^			1.000 (reference)	
	< 18.5 kg/m^2^			0.744 (0.621–0.891)	**0.001**
	25–29.9 kg/m^2^			1.164 (1.082–1.252)	**0.000**
	≥ 30.0 kg/m^2^			1.278 (1.102–1.483)	**0.001**
Tea consumption	no			1.000 (reference)	
	yes			0.885 (0.818–0.957)	**0.002**
Alcohol consumption	no			1.000 (reference)	
	yes			0.842 (0.768–0.923)	**0.000**
Life satisfaction	general	1.000 (reference)		1.000 (reference)	
	good	1.356 (0.987–1.863)	0.060	1.406 (1.014–1.951)	**0.041**
	poor	0.847 (0.775–0.925)	**0.000**	0.854 (0.780–0.934)	**0.001**
Experience of severe life events	no	1.000 (reference)			
	yes	1.109 (1.015–1.211)	**0.021**		
Feel isolated	no	1.000 (reference)		1.000 (reference)	
	yes	1.586 (1.253–2.009)	**0.000**	1.294 (1.089–1.538)	**0.003**
Feel ignored	no	1.000 (reference)			
	yes	0.796 (0.659–0.962)	**0.018**		
History of neck radiation	no	1.000 (reference)			
	yes	1.127 (1.002–1.269)	**0.047**		
TN family history	no	1.000 (reference)		1.000 (reference)	
	yes	1.108 (0.890–1.379)	0.361	1.334 (1.064–1.672)	**0.012**
	unknown	1.260 (1.117–1.422)	**0.000**	1.121 (0.990–1.270)	0.073
PSQI	good	1.000 (reference)			
	poor	1.168 (1.084–1.260)	**0.000**		
	no	1.000 (reference)			
frequent night shifts	yes	0.805 (0.721–0.898)	**0.000**		
Nap habit	no	1.000 (reference)		1.000 (reference)	
	throughout the year	0.952 (0.878–1.032)	0.230	1.044 (0.960–1.135)	0.313
	At certain seasons	1.087 (0.878–1.032)	**0.026**	1.150 (1.066–1.240)	**0.000**

Note: Values are expressed an adjusted odds ratio (95% CI). Bold fonts indicate statistically significant differences. Model 1: Potential risk factors (*p* < 0.05) from the chi-square (χ2) test were selected for multifactor logistic regression analysis (forward LR). Model 2: Based on Model 1, sociodemographic factors were adjusted for. The bolded values indicate a significant association between the variables. Abbreviations: BMI, body mass index; TN family history, thyroid nodule family history; PSQI, Pittsburgh Sleep Quality Index

## Discussion

Thyroid nodules are discrete lesions within the thyroid gland that are radiologically distinct from the surrounding thyroid tissue. Currently, thyroid ultrasound (US), a first-line diagnostic procedure, is widely used for detecting and characterizing nodular thyroid disease [26]. In this study, thyroid B-ultrasound was used to screen for thyroid nodules in adults in Zhejiang Province. The detection rate of thyroid nodules was 50.98%. With the increased use of high-resolution ultrasonography, a current prevalence of thyroid nodules greater than 60% has also been reported [27], and the thyroid nodule prevalence rate in this study was significantly lower than that reported by these authors. However, compared with the results of the national thyroid nodule prevalence survey in Zhejiang province in previous years, the detection rate of thyroid nodules has increased from 22.5 to 50.98% [28, 29]. The variability in prevalence between different global regions was probably related to differences in diagnostic practices, environmental exposures, individual risk factors, medical resources, health-care systems and national registries [4]. As for the increasing trend of thyroid nodule prevalence in Zhejiang province in recent years, the application of screening technology and the improvement of equipment and technical means can certainly explain part of the reasons. However, the influence of individual physical and mental conditions, family heredity, lifestyle, and individual exposure had also aroused our concern. After all, even if the deterioration rate of thyroid nodules is not high, a true increase in both the incidence and mortality of thyroid cancer has been supported by accumulating evidence [9]. Studies of thyroid cancer patients have shown that 46.1% of patients experience a psychological financial burden, and 28.1% of patients experience a material financial burden [30]. Among all cancer types, thyroid cancer is associated with the highest rate of bankruptcy [31]. Additionally, a meta-analysis revealed that 68.8% of all thyroid nodules undergoing surgical excision represented benign disease [32]. Surgical resection is widely chosen since both patients and clinicians are anxious about the risk of neoplasia [33]. This means that increasing the detection of benign and subclinical diseases may generate excess health care costs. It can be speculated that, if uncontrolled, the increasing prevalence of thyroid nodules would lead to serious economic and health threats to individuals and countries. Thus, prevention and treatment are both important strategies for the management of thyroid nodules. Research on the clinical diagnosis, classification and treatment of thyroid nodules is in progress, and the exploration of the causes of preventive thyroid nodules still needs the support of a large number of epidemiological investigations. In this study, we provide reference data to support this endeavor.

The chi-square analysis of the TN and no-TN groups in this study revealed significant intergroup differences in multiple indicators across four dimensions: sociodemographic factors (age, gender, education, BMI, smoking status, alcohol consumption status, and tea consumption status), psychosocial factors (life satisfaction, experience of severe life events, depressive mood, and social isolation), exposure and family history (history of neck radiation examination or treatment in the past three years, family history of thyroid nodules), and sleep status (PQSI index, nap habits, history of night shift work). However, in the binary multivariate logistic regression analysis, after adjusting for sociodemographic factors, only life satisfaction, social isolation, family history of thyroid nodules, and a habit of taking naps were associated with the risk of thyroid nodules. According to the study, poor life satisfaction is likly a risk factor for thyroid nodules (OR = 1.406, 95% CI: 1.014–1.951), whereas high life satisfaction is probably a protective factor against this condition (OR = 0.854, 95% CI: 0.780–0.934). With the advancement of psychosomatic medicine, the hypothalamus–pituitary–thyroid axis has garnered increasing attention. Studies have demonstrated that a fast-paced lifestyle, high-intensity work, and poor-quality sleep can exacerbate mental stress and emotional burden, leading to disruptions in the neuroendocrine system and resulting in psychosomatic disorders, including thyroid nodules [11, 14, 15, 34]. Life satisfaction refers to an individual’s overall evaluation of their physical and mental well-being. Numerous studies have suggested that poor life satisfaction is associated with adverse effects on physical health, mortality, and morbidity [19, 35, 36]. Poor life satisfaction is associated with a higher risk of thyroid nodules in this study, may serve as additional evidence of its adverse health effects. Generally, social isolation is accompanied by negative emotional experiences, and anxiety and depression are closely associated with thyroid diseases [37]. Therefore, the greater risk of thyroid nodules associated with social isolation (OR = 1.294, 95% CI: 1.089–1.538) may be mediated by emotional factors. Furthermore, this study suggested that a family history of thyroid nodules and a habit of napping at certain seasons are risk factors for thyroid nodules. In previous studies, it was reported that family history is a high risk factor for thyroid cancer [24, 38]. However, there are limited reports on the impact of family history on the risk of developing thyroid nodules. Whether this association is due to genetic regulation or shared living environments and family habits remains to be further investigated. The relationship between the risk of thyroid nodules and napping at certain seasons has not been previously reported. The fact that individuals only nap at certain seasons suggests that napping may not be a long-term personal habit but rather a bodily need arising from sleep deprivation or suboptimal physical and mental health during those periods. These factors, rather than napping itself, are likely the true underlying causes of the higher risk of thyroid nodules.

Gender, age, and BMI have been identified as risk factors for thyroid nodules in numerous domestic and international studies [39], and in this study, similar results were observed. Compared to males, females had a greater risk of developing thyroid nodules (TNs) (OR = 1.864, 95% CI: 1.727–2.011). Kung et al. [40] reported that pregnancies increased preexisting TNs and were also associated with the formation of new nodules, and the volume of benign TNs in most women after menopause did not change significantly [41]. Therefore, estrogen may be the cause of the higher incidence of TNs in women [42]. Since many morphological and even functional changes occur in the thyroid gland with age [43], it is not difficult to explain why the risk of thyroid nodules is positively related to age in this study. Compared with those of the 18- to 34-year-old group, the ORs of the 35- to 59-year-old group and the 60- to 69-year-old group were 1.606 and 3.060, respectively. In other words, aging is indeed a risk factor for TNs. The relationships between BMI and thyroid nodules were very close to those in the studies by Liang et al. [44], who reported that overweight status (OR = 1.164, 95% CI: 1.082–1.252) and obesity (OR = 1.278, 95% CI: 1.102–1.483), while a low BMI was a protective factor associated with lower rates of TN incidence (OR = 0.744, 95% CI: 0.621–0.891). This may be related to adipose tissue-mediated leptin secretion, as increased serum leptin levels in obese individuals can then contribute to TN development by promoting increased TSH levels [45]. Interestingly, the protective effect of a low BMI (< 18.5) against the risk of thyroid nodules has been reported many times, and the underlying mechanism of that association should be further investigated in the future. The protective effects of a high education level and regular tea consumption against the risk of thyroid nodules in this study are not difficult to understand because the group with a high education level also has relatively high health awareness. Tea polyphenols in tea have antioxidant capacity and may play an effective role in inhibiting reactive oxygen species and free radical pathways to damage cells, causing fibrosis and aging of thyroid tissue and ultimately leading to TN formation [46]. However, the fact that in this study, alcohol consumption was also a protective factor against thyroid nodules, and in previous reports [47, 48], alcohol consumption was shown to be a protective factor against thyroid cancer, is somewhat puzzling; this conclusion will be further investigated in the follow-up study of this cohort.

Compared with the results of univariate chi-square analysis, the associations of multiple different factors with thyroid nodules changed before or after adjustment for sociodemographic factors in multivariate regression analysis. Among the factors of smoking status, low mood, neck radiation, PSQI score and night shift status, some or all no longer entered the regression formulas of model, which was different from the findings of other relevant reports [11, 18, 49]. In addition to the differences in research methods and subjects, we must acknowledge the possible limitations of this study. First, the present study was based on a self-report questionnaire, which was inevitably affected by recall bias. Moreover, different recall times (such as neck radiation examinations in the past 3 years and depressive emotions only within the past month) caused different interference effects. Second, we aimed to explore the relationships between different influencing factors (including physiological, psychological and social factors) and the risk of thyroid nodules. Although the observation of social psychological factors was our focus, in this study, the choice of indicators was not comprehensive, novel or in-depth enough. Moreover, limited by the time required to answer the questionnaire, some observational dimensions used only concise questions instead of standard comprehensive structured questionnaires. Finally, this was a baseline survey of a cohort study involving cross-sectional observation; therefore, the observed associations may not be causal, and reverse causation cannot be ruled out. We will address the above limitations in subsequent cohort research. Specifically, we will adopt a thematic approach, such as the special topic of psychological factors and thyroid nodules, and further utilize standardized information collection tools while unifying the time frame for data gathering. By employing a prospective methodology, we will strictly control for various biases and oversights. Additionally, we will adopt innovative statistical methods for data analysis and exploration. This comprehensive approach will enable us to rigorously investigate the causal associations between various influencing factors and the risk of thyroid nodules in Zhejiang province. Ultimately, our objective is to provide reliable evidence for the scientific management of thyroid nodules, shifting the focus from posterior diagnosis and treatment to earlier prevention and detection.

## Conclusions

The results of thyroid ultrasonography in Zhejiang province indicate that the thyroid health status of the population warrants attention. Currently, the detection rate of thyroid nodules is 50.98%, which is increasing compared to that in previous years. In addition to the sensitive thyroid nodule screening technology, influcing factors mentioned in this study might also represent credible candidates for this increase. Advanced age, elevated BMI, female gender, low life satisfaction, isolated social status, and a family history of thyroid nodules all were risk factors of thyroid nodules. On the other hand, lower BMI, higher educational level, high life satisfaction, and long-term tea consumption appear to be protective against thyroid nodules. In this study, we provided valuable information for the scientific management of thyroid nodule prevention and health care.

### Electronic supplementary material

Below is the link to the electronic supplementary material.

Supplementary Material 1

## Data Availability

The datasets supporting the conclusions of this article are available from the corresponding author upon reasonable request.

## Abbreviations

OR: Odds Ratio
CI: Confidence Interval
TN: Thyroid nodules
ASRs: Age-standardized rates
BMI: Body mass index
PSQI: Pittsburgh Sleep Quality Index
