# Supplementary material for: Prevalence of thyroid nodule and relationship with physiological and psychosocial factors among adults in Zhejiang Province, China: a baseline survey of a cohort study
Source: BMC Public Health. 2024 Jul 11;24:1854. doi: 10.1186/s12889-024-19375-z (PMC11238450; doi:10.1186/s12889-024-19375-z)
Supplement: Supplementary file 1 — Supplementary Material 1 [file 12889_2024_19375_MOESM1_ESM.docx]

**Table S1. A summary of questionnair components: Zhejiang provincial natural population cohort study on environment and health (the part involved in this study)**

| **Individual questionnaire** | **Description** |
| --- | --- |
| sociodemographic characteristics | age, gender |
|  | What's your educational level?  [ ]junior high school and below [ ]high school [ ]university and above |
|  | Do you drinking tea? (No tea drinking or less than once a week is defined as no tea drinking)  [ ]Yes [ ]No |
|  | Do you drink alcohol? (No alcohol or less than once per month is defined as no drinking)  [ ]Yes [ ]No |
|  | Do you smoke? (Never smoked or less than once a month is defined as no smoking)  [ ]Yes [ ]No |
|  | BMI: Body mass index (BMI) was calculated as weight (kg) divided by height squared (m^2^). Height and weight were measured by uniform equipment. |
| Social psychological status | Are you satisfied with your life?  [ ]dissatisfied [ ]generally satisfied [ ]satisfied. |
|  | Have you experienced any of the following severe events during the past two years?  [ ]separated/divorced  [ ]unemployed/laid off/retired  [ ]economic bankruptcy of self-owned business or family  [ ]violent attacked/raped  [ ]serious internal family conflicts and clash  [ ]severe trauma or traffic accidents  [ ]death of spouse  [ ]other family members who died or become seriously ill  [ ]severe natural disasters (e.g., drought, flood, etc.)  [ ]loss of income/living in debt |
|  | Have you felt depressed in the last month?  [ ]Yes [ ]No |
|  | Have you felt nervous or anxious in the last month?  [ ]Yes [ ]No |
|  | Do you often feel the lack of company?  [ ]Yes [ ]No |
|  | Do you often feel be isolated by others?  [ ]Yes [ ]No |
|  | Do you often feel be ignored?  [ ]Yes [ ]No |

**Continue the above table**

| **Individual questionnaire** | **Description** |
| --- | --- |
| exogenous substance exposure and family history | Do you take any iodine supplements in addition to food and salt?  [ ]Yes [ ]No |
|  | Do you take any of the following medications in the last three months?  [ ]amiodarone  [ ]phenytoin sodium  [ ]rifampicin  [ ]estrogen  [ ]androgen  [ ]somatotropin  [ ]long-acting contraceptives  [ ]heparin |
|  | Have you received any of the following examinations or treatments for your head and neck in the past three years?  [ ]Radionuclide Co60 in the neck  [ ]I131 radiation in the neck  [ ]CT examination of the neck  [ ]magnetic resonance imaging of the neck |
|  | Did parents or siblings have a diagnostic history of thyroid nodules?  [ ]Yes [ ]No [ ]unclear |
| Sleep-related assessment | Pittsburgh sleep quality index, PSQI **(Table S2)** |
|  | In your current or previous job, did you often work night shifts?  [ ]Yes [ ]No |
|  | Do you have the habit of taking a nap?  [ ]Yes, all year. [ ]Yes, specific seasons [ ]No |
|  | Have you ever been diagnosed with obstructive sleep apnea?  [ ]Yes [ ]No |

**Table S2. Pittsburgh Sleep Quality Inde,PSQI**

| **order** | **Item** | **grade** | | | |
| --- | --- | --- | --- | --- | --- |
|  |  | 0 point | 1 point | 2 point | 3 point |
| 1 | Nearly a month, go to bed usually at o 'clock at night （24h system） | | | | |
| 2 | Nearly 1 month, it usually takes min from going to bed to falling asleepmin | □≤15min | □16~30min | □31~60min | □≥60min |
| 3 | Nearly 1 month, usually get up at o 'clock in the morning (24h system) | | | | |
| 4 | Nearly 1 month, usually actually slept h per night (not equal to duration in bed) | | | | |
| 5 | In the past one month, I was troubled by the following conditions affecting my sleep | | | | |
|  | a.Difficulty falling asleep (unable to fall asleep within 30 minutes) | □ never | □<1 time/week | □1~2 times/week | □≥3 times/week |
|  | b.Easy to wake up at night or early | □ never | □<1 time/week | □1~2 times/week | □≥3 times/week |
|  | c.Going to the bathroom at night | □ never | □<1 time/week | □1~2 times/week | □≥3 times/week |
|  | d.Can't breathe easily | □ never | □<1 time/week | □1~2 times/week | □≥3 times/week |
|  | e.Coughing or snoring loudly | □ never | □<1 time/week | □1~2 times/week | □≥3 times/week |
|  | f.Feel cold | □ never | □<1 time/week | □1~2 times/week | □≥3 times/week |
|  | g.Feel hot | □ never | □<1 time/week | □1~2 times/week | □≥3 times/week |
|  | h.Have a nightmare | □ never | □<1 time/week | □1~2 times/week | □≥3 times/week |
|  | i.Pain and discomfort | □ never | □<1 time/week | □1~2 times/week | □≥3 times/week |
|  | j.Other things that affect sleep: eg | □ never | □<1 time/week | □1~2 times/week | □≥3 times/week |
| 6 | In the past month, in general, how is your sleep quality? | □very good | □good | □poor | □very poor |
| 7 | In the past month, how often have you used drugs for hypnosis? | □ never | □<1 time/week | □1~2 times/week | □≥3 times/week |
| 8 | In the past month, do you often feel sleepy? | □ never | □<1 time/week | □1~2 times/week | □≥3 times/week |
| 9 | In the past month, how do you feel about your lack of energy? | □ never | □occasionally | □sometime | □often |

scoring method：

| **element** | **content** | **grade** | | | |
| --- | --- | --- | --- | --- | --- |
|  |  | **0 point** | **1 point** | **2 point** | **3 point** |
| A、subjective sleep quality | Item 6 was scored | □very good | □good | □poor | □very poor |
| B、sleep latency | The scores for items 2 and 5a were accumulated | □0 point | □1~2 points | □3~4 points | □5~6 points |
| C、sleep duration | Item 4 was scored | □>7h | □6~7h(excluding 6h) | □5~6h(including 6h) | □<5h |
| D、Sleep efficiency | Responses to items 1, 3, and 4 were used to calculate sleep efficiency* | □>85% | □75~85%(excluding75%) | □65~75%(including75%) | □<65% |
| E、Sleep disturbance | The scores of items 5b-5J were accumulated | □0 point | □1~9 points | □10~18 points | □19~27 points |
| F、use of sleep medication | Item 7 was scored | □ never | □<1 time/week | □1~2 times/week | □≥3 times/week |
| G、Days dysfunction due to sleepiness | The scores of items 8 and 9 were accumulated | □0 points | □1~2 points | □3~4 points | □5~6 points |

*Sleep efficiency calculation methods：

Sleep efficiency=$\frac{\text{item }4\text{（sleep duration）}}{\text{item }3(\text{wake up time)}-\text{item }1\text{（time to bed）}}$×100%

PSQI = A + B + C + D + E + F + G
